# Variation in tube voltage for pediatric neck 64VCT: Effect on radiation dose and image quality

**DOI:** 10.1371/journal.pone.0259772

**Published:** 2021-11-12

**Authors:** Li-Guo Chen, Ping-An Wu, Hsing-Yang Tu, Ming-Huei Sheu, Li-Chuan Huang

**Affiliations:** 1 Department of Medical Imaging, Hualien Tzu Chi Hospital, Buddhist Tzu Chi Medical Foundation, Hualien, Taiwan; 2 Department of Medical Imaging and Radiological Sciences, Tzu Chi University of Science and Technology, Hualien, Taiwan; Pisa University Hospital, ITALY

## Abstract

Exposure to ionizing radiation can cause cancer, especially in children. In computed tomography (CT), a trade-off exists between the radiation dose and image quality. Few studies have investigated the effect of dose reduction on image quality in pediatric neck CT. We aimed to assess the effect of peak kilovoltage on the radiation dose and image quality in pediatric neck multidetector-row CT. Measurements were made using three phantoms representative of children aged 1, 5, and 10 years, with tube voltages of 80, 100, and 120 kilovoltage peak (kVp); tube current of 10, 40, 80, 120, 150, 200, and 250 mA; and exposure time = 0.5 s (pitch, 0.984:1). Radiation dose estimates were derived from the dose-length product with a 64-multidetector-row CT scanner. Images obtained from the control protocol (120 kVp) were compared with the 80- and 100-kVp protocols. The effective dose (ED) was determined for each protocol and compared with the 120-kVp protocol. Quantitative analysis entailed noise measurements by recording the standard deviation of attenuation for a circular 1-cm^2^ region of interest placed on homogeneous soft tissue structures in the phantom. The mean noise of the various kVp protocols was compared using the unpaired Student t-test. Reduction of ED was 37.58% and 68.58% for neck CT with 100 kVp and 80 kVp, respectively. The image noise level increased with the decrease in peak kilovoltage. Noise values were higher at 80 kVp at all neck levels, but did not increase at 100 kVp, compared to 120 kVp in the three phantoms. The measured noise difference was the greatest at 80 kVp (absolute increases<2.5 HU). The subjective image quality did not differ among the protocols. Thus, reducing voltage from 120 to 80 kVp for neck CT may achieve ED reduction of 68.58%, without compromising image quality.

## Introduction

The optimization of computed tomography (CT) parameters involves a balance between image quality required for accurate diagnosis and radiation dose exposure. CT examination of the neck extends from the lower portion of the brain to the upper part of the chest. Exposure to ionizing radiation is associated with the risk of cancer, particularly in children, who tend to be more radiosensitive than adults [1–4]. Three recent studies from the UK, Netherlands, and Japan have published data supporting the association of CT performed in early childhood with the subsequent real risk of malignancy, which was attributed solely to the CT scan [1, 5, 6]. Pearce et al. [1] reported a positive association between the radiation dose from CT and leukemia [excess relative risk (ERR) per mGy: 0.036, 95% CI: 0.005–0.120] and brain tumors (ERR: 0.023, 95% CI: 0.010–0.049): pediatric CT scans delivering cumulative doses of about 50 mGy may almost triple the risk of leukemia and doses of about 60 mGy may triple the risk of brain cancer. The cumulative absolute risks are small because these cancers are relatively rare; one excess case of leukemia and one excess case of brain tumor per 10,000 head-CT scans are estimated to occur over 10 years after the first scan for patients younger than 10 years. Meulepas et al. [5] reported a positive association between the radiation dose from pediatric CT and the risk of cancer. The average cumulative brain dose of 38.5 mGy was (statistically) significantly associated with the risk for malignant and nonmalignant brain tumors combined (ERR/100 mGy: 0.86, 95% confidence interval = 0.20 to 2.22, P = 0.002; 84 cases). The incidence of brain tumors was higher in the cohort of children who underwent CT scans compared to the general population. Kadowaki et al. [6] investigated 138532 head CT examinations performed between the ages of 0 to 10 years. They found that CT consequently induced a lifetime excess of 22 cases (1 per 6300 scans) of brain/CNS cancers, accounting for 5% of the total cases.

The current protocol for dose reduction for neck multidetector-row computed tomography (MDCT) includes the placement of bismuth shields over the thyroid region and cervical spine collars. These shields may help achieve a dose reduction of 22.5% and 10.4% to the thyroid gland and cervical spine, respectively [7]. Moreover, Karmazyn et al. [8]. showed that lowering tube voltage to 80 kilovoltage peak (kVp) and 100 kVp may be effective for imaging in infants and children, respectively. However, reducing the peak voltage and using a fixed tube current is rare for pediatric CT in clinical practice, although this may help protect the neck, which is smaller than the chest and abdomen.

Radiation dose reduction must always be balanced with image quality. Using a lower peak kilovoltage for neck MDCT may decrease radiation dose and improve image quality. Improved image quality may be achieved with iodine-based contrast media, because imaging is closer to the K-edge of iodine [9, 10].

Another approach to radiation dose reduction involves lowering the tube voltage from the standard value of 120 kVp to 100 or 80 kVp, where the applied quality reference tube current-time product values are in the range of 45–680 mAs. Lowering the tube voltage confers the additional advantage of higher attenuation for iodinated contrast medium at lower X-ray tube voltages, given the greater photoelectric effect and decreased Compton scattering values [9]. However, the disadvantage of low tube voltage CT scanning is the concurrent increase in image noise, particularly when the tube current-time product is not suitably increased [11, 12]. Although pediatric neck CT examinations are performed frequently in clinical practice, the number of studies in this population is scarce [13, 14]. Thus, CT examination must be clinically indicated, with a high benefit-to-risk ratio. The CT acquisition protocol should be optimized to the region of imaging at the lowest possible radiation dose, in accordance with the “as low as reasonably achievable” (ALARA) principle.

Based on the available literature, we hypothesized that the protocol with 80 kVp combined with fixed tube current would result in dose limitation without compromising the image quality. The purpose of this study was to assess the feasibility, image quality, and radiation dose associated with low kilovoltage CT of the neck at a tube potential of 80 kVp.

## Materials and methods

### Experimental protocols for anthropomorphic prostheses

**Fig 1:**
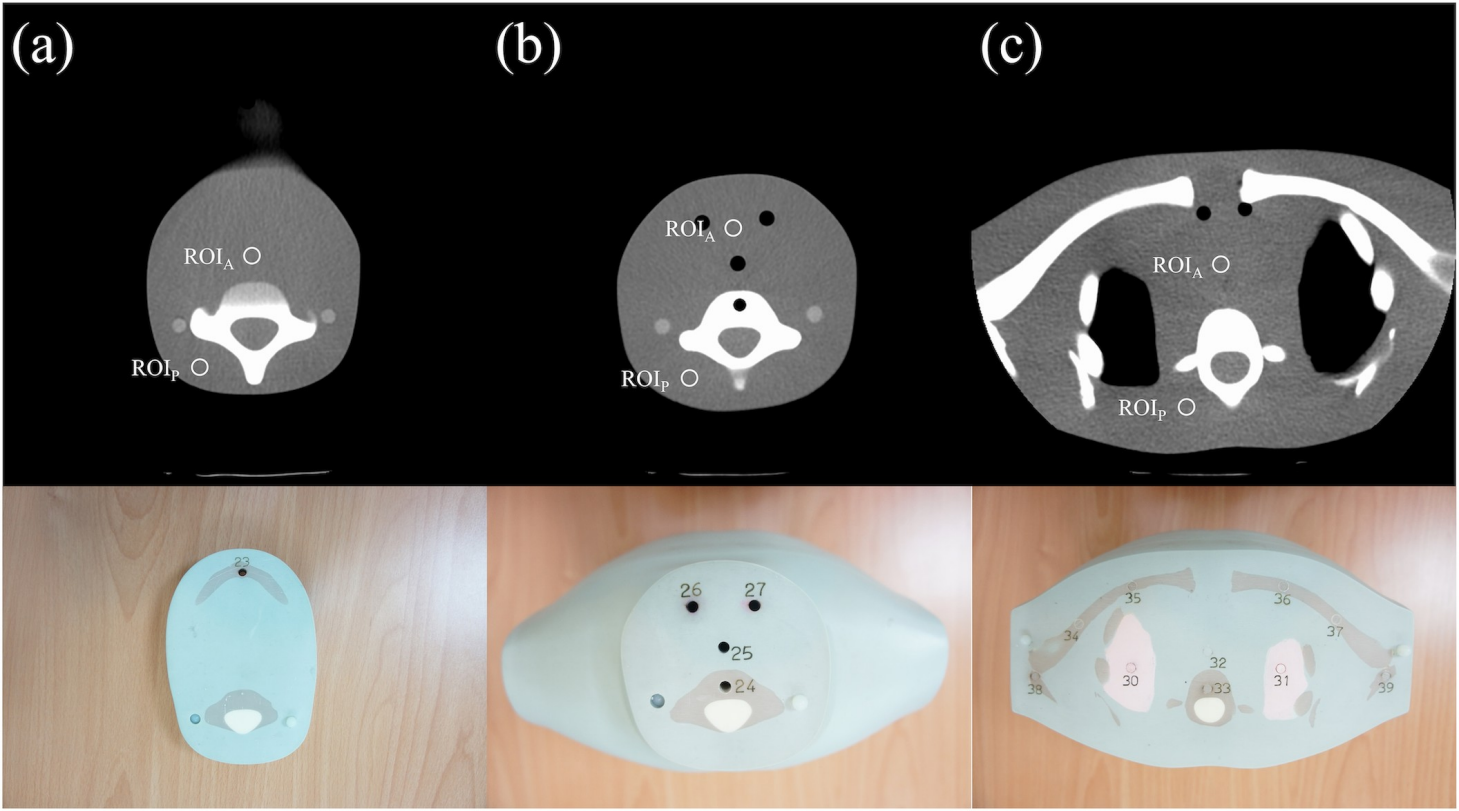
Computed tomography scans of phantom 10-year-old child. Three slice levels at which noise measurements were obtained (window width/level = 400/40): (a) upper neck, (b) middle neck, (c) lower neck and upper mediastinum. ROI, region of interest.

Three anthropomorphic prostheses (APs) (ATOM; CIRS, Norfolk, VA), representative of children aged 1, 5, and 10 years were used (Fig 1), consisting of 22-, 26-, and 32-numbered, 2.54-cm-thick transverse sections, respectively. CT images of three AP necks were acquired with a 64-section multidetector-row CT scanner (LightSpeed VCT; GE Healthcare, Inc, Milwaukee, WI) using successive scanning tube voltages of 80, 100, and 120 kVp. Each tube voltage was accompanied by tube current values of 10, 40, 80, 120, 150, 200, or 250 mA. Twenty-one CT scans were acquired with the three tube voltages and seven different values of tube current for each anthropomorphic phantom. The scanner configuration was 64 x 0.625 mm (gantry rotation time, 0.5 s; beam pitch, 0.984:1). The limited volume scan covered the following anatomic areas: the centrum semiovale, corona radiata at the lateral ventricles, middle cranial fossa/skull base, and posterior fossa/palate. This study was limited to CT examinations for general brain diseases such as brain edema, infection, injury, tumors, and cerebral hemorrhage.

The slice thickness was fixed at 2.5 mm for image reconstruction at 21 different combinations. The CT images were reconstructed using the standard reconstruction filter in 512 x 512 matrices with a pixel size of 1.367 mm. Subsequently, the volume CT dose index (CTDI_vol_: mGy), dose-length-product (DLP: mGy∙cm), effective dose (ED: mSv), and noise levels were estimated.

### Region of interest, CT number, and noise

Image quality was assessed using ImageJ (US National Institutes of Health, Bethesda, MD, USA; http://imagej.nih.gov/ij, version 1.51p). Noise measurements for quantitative analysis entailed recording the noise [Hounsfield units (HU]) of attenuation for a circular 1-cm^2^ region of interest placed on homogeneous soft tissue structures in the phantom. Noise levels were measured in the soft tissues in the anterior and posterior aspects of the neck at three selected locations along the z-axis of the neck. The levels encompassed the upper neck (immediately below the mandible), thyroid gland, and lower neck–upper mediastinum (Fig 1). The noise values per location at each level were averaged to acquire the mean noise level for the upper, mid, and lower neck and mediastinum.

### Dosimetry estimation

The radiation dose from the CT scans was calculated from the CTDI_vol_ and DLP. The CTDI_vol_ and DLP were displayed on the scanner’s console after each scan. The product of CTDI_vol_ and the scan length provided the DLP related to the total amount of energy deposited in the patient. The ED is the product of DLP and the *k* factor; the *k* factor used was described in the ICRP-103 report by the International Commission on Radiological Protection (ICRP) [15].

The CTDI_vol_ is the average radiation dose in the scanned area, measured in a standard acrylic cylinder with a 16-cm test phantom diameter. The weighted DLP is the product of CTDI_vol_ and the length (d) of the scanned area. The latest scanners usually provide dose displays for both CTDI_vol_ and DLP. Regular measurements verify the accuracy of the dose display reading as a part of scanner quality control (Eq. 1).


DLP(mGy∙cm)=CTDI(mGy)·d(cm) (1)

The ED for the neck was calculated by multiplying DLP, the individual dose report, with the dose conversion factor value (*k*) in mSv/mGy∙cm (Eq. 2), as recommended.

ED(mSv)=DLP(mGy·cm)×k(mSv/mGy·cm) (2)

where, the scan length weighting factors (*k*) for 80 kVp were 0.0171, 0.0123, and 0.0095 mSv/mGy∙cm, respectively; those for 100 kVp were 0.0167, 0.0121, and 0.0093 mSv/mGy∙cm, respectively; and those for 120 kVp were 0.0166, 0.012, and 0.0094 mSv/mGy∙cm, respectively, for the neck of APs simulating children aged 1, 5, and 10 years [15]. The relationships among CTDI_vol_, DLP, ED and noise values per age group are represented by Eqs (1) and (2).

### Statistical analysis

Statistical analysis was performed using GraphPad Prism 6 (Graphpad Software Inc., La Jolla, CA, USA) software. The mean EDs for the 100- and 80-kVp protocols were compared with those of the reference 120-kVp protocol using two-factor analysis of variance with all possible pairwise interactions. The unpaired Student t-test was used to compare the mean noise values of the 100- and 80-kVp protocols with those of the reference 120-kVp protocol. Two-tailed p-values <0.05 were considered statistically significant.

## Results

The CTDI_vol_ and DLP obtained using the three anthropomorphic phantoms and 21 combinations of kVp and mA were in the range 0.26–21.27 mGy and 4.25–465.42 mGy-cm, respectively. At the same kVp and mA setting, an increase in the DLP corresponding to the anthropomorphic phantom simulating an older child was caused by extended scan length. DLP-based estimates of the ED were in the range of 0.05–5.96 mSv. The corresponding increase in ED was due to reduced attenuation and the change in the distribution of radiosensitive organs within the body in the AP simulating a younger child (Table 1).

**Table 1:** Effective dose in anthropomorphic prostheses-based neck computed tomography scans, representing children aged 1, 5, and 10 years.

Tube voltage (kVp)	Tube current (mA)	CTDI_VOL_ (mGy)	1-year-old		5-year-old		10-year-old	
			DLP	Effective dose	DLP	Effective dose	DLP	Effective dose
			(mGy-cm)	(mSv)	(mGy-cm)	(mSv)	(mGy-cm)	(mSv)
80	10	0.26	4.25	0.07	4.93	0.06	5.77	0.05
	40	1.05	17.81	0.30	19.90	0.24	23.08	0.22
	80	2.11	35.62	0.61	39.98	0.48	46.17	0.44
	120	3.16	53.43	0.91	59.88	0.72	69.25	0.66
	150	3.96	66.79	1.14	75.04	0.90	86.57	0.82
	200	5.27	89.50	1.53	99.87	1.20	115.42	1.10
	250	6.59	111.31	1.90	124.88	1.50	144.28	1.37
100	10	0.53	8.90	0.15	10.04	0.12	11.54	0.11
	40	2.11	35.62	0.59	39.98	0.48	46.17	0.43
	80	4.22	71.24	1.19	79.97	0.97	92.34	0.86
	120	6.33	106.86	1.78	119.95	1.45	138.51	1.29
	150	7.91	133.57	2.23	149.89	1.81	173.14	1.61
	200	10.55	178.10	2.97	199.92	2.42	230.85	2.15
	250	13.19	222.62	3.72	249.95	3.02	288.56	2.68
120	10	0.85	14.36	0.24	16.11	0.19	18.62	0.18
	40	3.40	57.45	0.95	64.43	0.77	74.47	0.70
	80	6.81	114.90	1.91	129.05	1.55	148.93	1.40
	120	10.21	172.35	2.86	193.48	2.32	223.40	2.10
	150	12.76	215.44	3.58	241.80	2.90	279.25	2.62
	200	17.02	287.26	4.77	322.53	3.87	372.34	3.50
	250	21.27	359.07	5.96	403.07	4.84	465.42	4.37

Table 1 shows the ED calculated from the estimated CT dose index and DLP. All values decreased with the reduction in peak kilovoltage. The ED decreased by 68.12% and 37.58% with the 80-kVp and 100-kVp protocols, respectively, relative to that of the 120-kVp protocol.

### Image quality assessment

**Fig 2:**
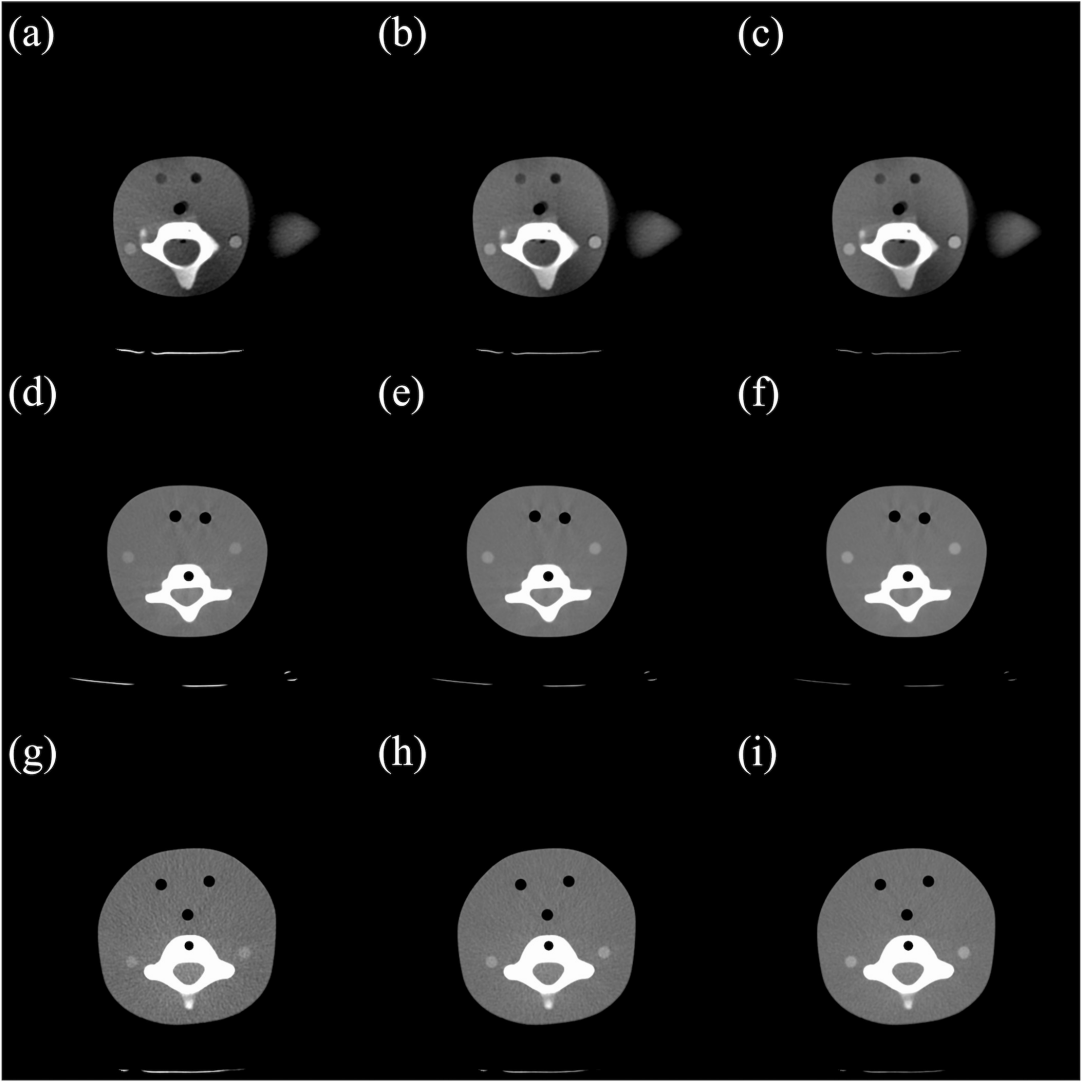
Computed tomography images of the phantom at the level of the upper neck obtained with different voltage protocols. The difference in the measured image noise was the greatest at this level: (a) 1-, (d) 5-, and (g) 10-year-old, 80 kVp; (b) 1-, (e) 5- and (h) 10-year-old, 100 kVp; (c) 1-, (f) 5-, and (i) 10-year-old, 120 kVp.

In the simulations of the 1-year-old child’s scan, noise values were higher for the 80-kVp protocol than that for the 120-kVp protocol at all 3 levels of the neck; however, the absolute differences in image noise were less than 3–4 HU (Table 2). The subjective assessment of image quality revealed no differences in the quality of images obtained by the 80 kVp protocol compared to those obtained with the 120-kVp protocol. Lowering the tube voltage to 100 kVp did not increase the noise values in the mid or lower neck. However, a statistically significant increase in noise values was observed in the upper neck. The absolute difference in image noise at this level was only 1–2.2 HU. The subjective assessment of image quality revealed no differences in the quality of images obtained with the 80- and 100-kVp protocols compared to those obtained with the 120-kVp protocol (Fig 2).

**Table 2:** Results of objective analyses of reconstruction techniques for computed tomography imaging of simulated neck scans of a child aged 1 years.

Protocol	Anterior upper neck		Posterior upper neck		Anterior thyroid gland		Posterior thyroid gland		Anterior upper mediastinum		Posterior upper mediastinum	
	CT number (HU)	Noise (HU)	CT number (HU)	Noise (HU)	CT number (HU)	Noise (HU)	CT number (HU)	Noise (HU)	CT number (HU)	Noise (HU)	CT number (HU)	Noise (HU)
**80 kVp**												
10 mA	8.65	31.32	6.32	26.33	10.56	29.49	-0.67	30.18	15.77	40.01	19.56	59.72
40 mA	1.16	16.17	4.78	12.53	-1.97	17.10	10.06	16.46	14.89	20.66	12.04	24.41
80 mA	24.61	13.06	20.59	10.26	-13.96	10.26	-22.97	12.80	9.19	13.66	12.56	16.06
120 mA	2.60	9.40	6.32	8.50	0.58	10.36	-6.80	11.10	13.28	10.35	12.87	12.91
150 mA	2.00	9.49	5.91	7.46	0.67	9.26	-10.10	11.04	13.12	9.43	12.34	12.18
200 mA	27.50	8.53	20.63	7.10	-15.81	8.13	-19.78	9.44	12.11	8.37	12.60	10.00
250 mA	16.92	7.17	21.72	6.88	-15.24	6.44	-17.64	8.64	13.03	8.13	12.69	9.49
**100 kVp**												
10 mA	4.61	20.76	10.31	20.09	2.82	23.62	-4.96	21.61	17.70	28.75	13.15	35.29
40 mA	28.92	14.09	25.17	9.46	-12.72	10.45	-12.53	11.69	15.19	12.33	16.08	17.01
80 mA	7.00	8.98	10.82	7.06	4.64	9.68	-5.59	9.92	16.98	11.44	17.57	12.52
120 mA	29.43	8.06	16.04	6.01	-9.79	6.07	-13.21	8.36	15.55	9.02	17.28	9.87
150 mA	7.10	6.67	10.32	5.48	3.29	7.91	-6.95	7.47	17.20	8.76	15.35	9.13
200 mA	7.40	5.93	11.32	5.40	1.07	7.82	-5.74	6.78	16.87	7.34	17.43	8.59
250 mA	8.02	5.27	10.98	5.36	4.10	6.69	-0.27	6.72	18.77	6.87	17.39	7.62
**120 kVp**												
10 mA	28.99	20.67	26.56	17.34	-12.74	17.76	-10.31	19.85	18.17	22.59	17.26	28.98
40 mA	10.03	9.66	12.50	8.55	6.10	11.09	-3.33	11.06	19.68	12.83	20.10	12.67
80 mA	10.37	7.18	13.66	6.00	3.32	8.54	-0.98	7.67	19.46	9.64	19.87	11.50
120 mA	10.97	6.95	14.46	5.80	0.93	8.10	-1.35	7.39	19.07	7.10	19.63	7.85
150 mA	11.04	5.67	13.66	5.35	0.92	7.05	-1.98	7.07	20.04	6.52	20.48	7.48
200 mA	33.40	5.51	28.13	5.13	-9.08	5.45	-6.44	6.94	18.33	5.26	19.47	6.26
250 mA	10.74	3.79	14.32	4.56	12.26	5.14	-1.33	6.00	20.42	4.76	19.78	4.87
Comparison	*p*											
**120 kVp vs. 100 kVp**		NS		NS		NS		NS		NS		NS
**120 kVp vs. 80 kVp**		NS		NS		NS		NS		NS		NS

The noise values were higher for the 80-kVp protocol than for the 120-kVp protocol at all three levels of the neck in the 5-year-old child’s scanning simulations. However, the absolute differences in the image noise values were less than 3–4.4 HU (Table 3). Lowering the tube voltage to 100 kVp did not increase noise values obtained in the mid, upper, or lower neck. At this level, the absolute difference in the image noise values was only 1–2.2 HU. The subjective assessment of image quality revealed no differences in the quality of images obtained with the 80- and 100-kVp protocols compared to those obtained with the 120-kVp protocol (Fig 2).

**Table 3:** Results of objective analyses of reconstruction techniques for computed tomography imaging of simulated neck scans of a child aged 5 years.

Protocol	Anterior upper neck		Posterior upper neck		Anterior thyroid gland		Posterior thyroid gland		Anterior upper mediastinum		Posterior upper mediastinum	
	CT number (HU)	Noise (HU)	CT number (HU)	Noise (HU)	CT number (HU)	Noise (HU)	CT number (HU)	Noise (HU)	CT number (HU)	Noise (HU)	CT number (HU)	Noise (HU)
**80 kVp**												
10 mA	16.74	20.09	14.27	16.21	18.24	16.54	14.31	13.67	24.09	44.05	16.01	38.32
40 mA	11.21	9.62	14.45	8.71	16.77	9.34	16.35	7.91	21.96	15.13	12.70	14.98
80 mA	12.05	7.27	16.51	7.22	18.09	6.02	17.62	6.13	20.93	10.80	13.67	10.59
120 mA	12.25	5.93	15.10	5.64	18.54	5.86	17.62	5.25	22.42	8.42	14.14	9.81
150 mA	13.07	5.43	14.82	5.17	18.85	5.40	16.39	4.81	20.80	7.19	15.32	8.32
200 mA	11.94	4.20	14.89	4.87	18.05	4.34	16.43	4.78	24.59	6.98	16.49	7.82
250 mA	13.24	4.00	16.23	4.67	18.43	4.25	17.85	3.96	20.49	6.75	14.29	7.46
**100 kVp**												
10 mA	17.12	14.33	19.06	10.98	21.99	11.96	20.14	11.32	21.81	20.55	15.73	18.99
40 mA	15.84	6.47	18.51	6.14	22.31	5.92	19.29	5.86	22.55	9.89	17.75	9.73
80 mA	16.77	4.66	18.05	4.66	21.30	4.53	18.92	4.52	23.05	7.18	18.05	8.92
120 mA	16.56	4.70	18.19	4.50	22.89	4.30	19.73	3.96	22.08	5.85	17.74	6.38
150 mA	16.87	3.50	19.27	4.26	22.60	4.24	19.38	3.65	19.45	5.68	18.60	5.94
200 mA	16.68	3.07	19.06	3.72	22.57	4.18	18.62	3.40	24.57	4.83	19.65	5.35
250 mA	16.42	3.01	18.90	3.45	22.45	4.09	18.01	3.01	25.01	4.34	18.99	5.01
**120 kVp**												
10 mA	19.57	11.01	21.05	9.45	24.61	9.72	22.24	9.14	23.80	15.79	18.33	17.25
40 mA	18.75	5.49	21.69	5.62	24.99	5.09	21.64	4.42	23.42	8.86	20.64	7.89
80 mA	19.00	4.00	19.71	4.44	23.80	4.26	22.54	3.73	26.58	6.36	20.63	6.88
120 mA	19.26	3.61	19.41	4.26	24.92	4.19	21.23	3.41	25.08	5.52	19.58	6.03
150 mA	20.33	2.59	21.04	3.86	25.58	4.08	21.39	3.40	26.02	4.69	21.51	4.85
200 mA	20.07	2.46	21.32	3.43	25.37	4.03	21,90	3.17	22.59	4.61	21.32	4.45
250 mA	20.01	2.30	22.59	3.26	24.37	3.22	22.01	2.84	26.06	3.97	22.26	3.88
Comparison	*P*											
**120 kVp vs. 100 kVp**		NS		NS		NS		NS		NS		NS
**120 kVp vs. 80 kVp**		NS		NS		NS		NS		NS		NS

The noise values were higher for the 80-kVp protocol than that for the 120-kVp protocol at all three levels of the neck in the 10-year-old child’s scanning simulations. The absolute differences in image noise values were less than 3–18 HU (Table 4). The subjective assessment of image quality revealed no differences in the quality of images obtained with the 80 kVp protocols compared to those obtained with the 120-kVp protocol. Lowering the tube voltage to 100 kVp did not increase noise values of the mid or upper neck, but there was a statistically significant increase in noise values in the lower neck. The absolute difference in image noise at this level was only 2–7 HU. The subjective assessment of image quality revealed no differences in the quality of images obtained with the 80, 100, and 120-kVp protocols (Fig 2).

**Table 4:** Results of objective analyses of reconstruction techniques for computed tomography imaging of simulated neck scans of a child aged 10 years.

Protocol	Anterior upper neck		Posterior upper neck		Anterior thyroid gland		Posterior thyroid gland		Anterior upper mediastinum		Posterior upper mediastinum	
	CT number (HU)	Noise (HU)	CT number (HU)	Noise (HU)	CT number (HU)	Noise (HU)	CT number (HU)	Noise (HU)	CT number (HU)	Noise (HU)	CT number (HU)	Noise (HU)
**80 kVp**												
10 mA	39.66	87.93	33.80	63.14	21.97	53.66	23.10	62.73	13.04	139.25	60.59	149.87
40 mA	21.37	25.87	15.30	26.08	16.10	22.37	25.64	24.54	21.88	51.03	20.74	49.60
80 mA	20.17	17.39	13.20	20.47	16.51	14.85	20.12	17.37	18.96	32.96	18.52	32.79
120 mA	23.12	15.99	13.85	15.58	15.20	13.29	19.73	14.64	20.49	26.32	20.18	28.15
150 mA	19.99	11.58	12.62	13.29	17.74	9.81	22.45	14.37	24.50	21.77	12.31	24.83
200 mA	21.57	9.98	12.92	12.84	14.68	8.82	22.00	10.34	20.26	17.75	17.96	22.67
250 mA	21.48	9.78	16.22	11.51	18.25	7.64	18.96	10.18	21.46	17.31	18.19	18.49
**100 kVp**												
10 mA	25.84	43.28	14.95	38.58	17.09	31.26	25.23	32.93	16.21	65.71	40.87	78.50
40 mA	24.10	16.93	19.20	18.48	20.64	15.83	23.33	18.96	17.71	29.95	18.00	33.09
80 mA	25.51	13.77	15.61	12.40	19.53	10.73	25.36	12.18	19.52	17.84	20.90	21.66
120 mA	21.78	9.87	17.89	10.13	21.08	9.60	26.00	10.63	19.40	15.88	18.06	18.41
150 mA	25.40	8.78	16.81	8.93	20.69	8.07	23.97	9.54	18.72	12.30	20.43	15.73
200 mA	23.23	7.71	18.33	8.46	21.50	7.67	27.75	8.35	18.80	12.01	16.73	12.31
250 mA	25.10	6.91	16.94	8.02	20.27	7.05	24.88	6.96	20.28	11.92	21.10	11.21
**120 kVp**												
10 mA	25.20	26.18	21.82	26.42	21.65	23.86	27.99	26.52	26.20	48.29	21.68	48.57
40 mA	27.07	15.58	17.79	15.52	23.88	13.98	26.11	13.04	22.12	20.80	22.25	22.31
80 mA	27.46	10.58	19.97	10.08	23.33	8.82	25.40	9.83	21.59	18.87	23.13	14.54
120 mA	25.27	7.53	21.66	9.14	23.49	6.70	29.95	8.21	22.87	14.51	19.99	13.18
150 mA	25.50	7.41	22.16	8.16	24.10	6.65	29.41	8.10	22.16	11.29	19.65	12.91
200 mA	26.25	6.51	21.17	7.45	24.11	5.37	28.59	7.65	23.62	10.49	20.46	10.55
250 mA	27.37	6.13	20.31	6.76	22.57	5.24	27.65	6.60	21.02	9.83	23.53	8.90
Comparison	*P*											
**120 kVp vs. 100 kVp**		NS		NS		NS		NS		NS		NS
**120 kVp vs. 80 kVp**		NS		NS		NS		NS		NS		NS

## Discussion

To the best of our knowledge, this study is the first to evaluate the effect of variable peak kilovoltage used in conjunction with a fixed tube current on image quality in pediatric neck MDCT. This phantom study simulated image acquisition in children aged 1, 5, and 10 years, showing that reducing the peak kilovoltage while using a fixed current reduced the effective radiation dose for neck MDCT protocols without an increase in image noise and decrease in image quality.

This study used three types of anthropomorphic models and fixed rather than automatic tube current modulation. We found that 80-kVp and 100-kVp protocols were associated with an ED reduction of 68.58% and 38.03%, respectively, relative to that achieved with the standard 120-kVp protocol. The dose reductions obtained in the present study were smaller than previously reported values [16].

While designing the present study, we considered that automatic tube current modulation might nullify the dose savings associated with lower peak kilovoltage due to the high upper limit of the tube current. We hypothesized that using 80 kVp in combination with a fixed tube current would result in dose savings without compromising the image quality.

The dose reduction was 68.58% with a lower peak kilovoltage. This dose saving may help reduce the stochastic risk of carcinogenesis from radiation during MDCT of the neck [4, 17]. A chohrt study has shown the relative risk of brain tumor for individuals who received a cumulative dose of 50–74 mGy was up to 2.82 compared with individuals who received an amount of less than 5 mGy [1]. Another study that used the blood dose to analyze the dose-response of the head and neck regions in adults showed that CT of the head and neck region provides a sufficiently high radiation dose (CTDI_vol_: 25.26–44.21 mGy and DLP: 446.25–905.08 mGy-cm) to induce double-strand breaks in the DNA of cells in the peripheral blood. The CTDIvol in our study was 0.26–21.27 mGy, and the DLP was 4.25–465.42 mGy-cm, both of which were lower than previous studies [18].

Using optimal tube potential may help reduce radiation dose and improve workflow [10]. The objective and subjective image quality of neck CT images acquired at different tube current-time products (275 mAs and 340 mAs) and reconstructed with filtered-back-projection (FBP) and adaptive statistical iterative reconstruction were analyzed in this study. Adaptive statistical iterative reconstruction in neck CT protocols reduces the ED by 17% [19]. Meanwhile, Yu et al. [10] reported that for the 10-cm phantom, the radiation dose is reduced by 12% at 80 kVp and by 8% at 100 kVp, relative to the dose delivered at 120 kVp. When the noise level matches an image obtained at 120 kVp, the likelihood of dose reduction at lower tube potentials is limited or non-existent.

Placing bismuth shields on the skin during pediatric CT scanning may reduce the radiation dose to the thyroid gland and chest, without inducing deterioration of the image quality. Inkoom et al. [20] examined the effect of bismuth shielding on the thyroid dose and image quality during pediatric neck CT performed with a fixed tube current (FTC) and automatic exposure control (AEC) using a 16-slice MDCT system and four pediatric anthropomorphic phantoms. They found that the FTC and single-layered bismuth shielding did not affect the thyroid dose considerably. AEC was more effective in thyroid dose reduction than the in-plane bismuth shields. The application of cotton spacers in the bismuth shield had no impact on the thyroid dose, but significantly decreased the image noise.

The effect of CT dose reduction techniques on image quality is an important consideration. The absolute differences in noise values between the 80-kVp and reference protocols were slight in the present study and below the readers’ threshold of detection. These findings are similar to those of Masuda et al. [21], who showed that the image noise was significantly higher for the 80-kVp protocol (mean noise, 40 HU) than that for the 120-kVp protocol (mean noise, 25 HU). However, these differences were not evident on our subjective assessment. Higaki et al. [22] demonstrated that the noise commonly encountered on CT images can affect diagnostic accuracy. They developed a deep-learning reconstruction (DLR) method that integrated deep convolutional neural networks into image reconstruction to reduce the image noise. The image noise was lower with the images obtained with DLR method compared to the FBP images, and the high-contrast spatial resolution and task-based detectability were better than those of images reconstructed with other state-of-the-art techniques.

Several enhanced dose-reduction strategies can be used to optimize scan protocols and reduce patient dose, including using localizer images to optimize the kVp and mAs and to customize the scan to an individual patient’s anatomy. The use of a decreased kVp in children can reduce the radiation dose and may improve soft-tissue contrast [23]. While a fixed tube current was used in this study, dose modulation was also used correctly. With correct selection of the minimum and maximum mA values and noise index or similar image quality factors, one can determine a dose similar to that with a fixed current. Faster computers facilitate more sophisticated image reconstruction algorithms, such as iterative reconstruction, and improved image quality, while permitting the use of a reduced mAs and, therefore, reduced-dose imaging. Reconstruction of virtual monoenergetic imaging (VMI) series has beneficial effects for non-contrast and contrast-enhanced dual-energy CT (DECT) due to the flexibility in calculating low-keV VMI reconstructions to increase contrast and iodine attenuation or to compute high-keV VMI reconstructions to reduce beam-hardening artifacts [24]. The low-keV VMI and iterative reconstructions may help in dose reduction and in improvement of image contrast in modern scanners.

There were several limitations to our study. First, the parameter limit involved a fixed dose with a noise index of the maximum tube current of 250 mA and the minimum tube current of 10 mA. The tube current value for adult CT scanning (500 mA) established by Hoang et al. [25] was not reached in any of the protocols. The maximum tube currents reached in the 80-, 100-, 120-, and 140-kVp protocols were 431, 219, 120, and 100 mA, respectively. Removing or altering these factors could have improved the generalizability of our findings; however, we chose these limits because they are a part of our standard clinical protocol; this study aimed to evaluate the effect of peak kilovoltage on the absorbed doses in our clinical protocol.

Second, we analyzed only CT images obtained with a single type of MDCT scanner, which was provided by one manufacturer. The results may vary for other generations of CT scanners. Third, the data for dose comparisons between the FBP protocols were obtained from the measurements obtained with three anthropomorphic phantoms representative of children aged 1, 5, and 10 years with tube voltage values of 80, 100, and 120 kVp; tube current values of 10, 40, 80, 120, 150, 200, or 250 mA; and exposure time of 0.5 s at 0.984:1 pitch.

Finally, radiation dose estimates were derived from the DLP with a 64-MDCT scanner to assess the extent of radiation exposure with respect to the image quality. FBP protocols must be optimized, as required by the present study. Studies on FBP may help improve its application in clinical practice. However, we did not consider the minimum CT radiation dose that provides adequate anatomic correlation with the neck scan.

## Conclusion

Our phantom study showed that reducing peak kilovoltage and maintaining fixed neck MDCT reduced the effective radiation dose. A reduction in voltage from 120 to 80 kVp reduced the dose by 68.58%, without a decline in the subjective image quality.
